# Innovative Modeling of IMU Arrays Under the Generic Multi-Sensor Integration Strategy

**DOI:** 10.3390/s24237754

**Published:** 2024-12-04

**Authors:** Benjamin Brunson, Jianguo Wang, Wenbo Ma

**Affiliations:** Department of Earth and Space Science and Engineering, Lassonde School of Engineering, York University, Toronto, ON M3J 1P3, Canada; jianguo.wang@lassonde.yorku.ca (J.W.); mikewbmaster@gmail.com (W.M.)

**Keywords:** IMU array, Generic Multi-Sensor Integration Strategy, error analysis in discrete Kalman filtering, integrated kinematic positioning and navigation, Variance Component Estimation in discrete Kalman filtering

## Abstract

This research proposes a novel modeling method for integrating IMU arrays into multi-sensor kinematic positioning/navigation systems. This method characterizes sensor errors (biases/scale factor errors) for each IMU in an IMU array, leveraging the novel Generic Multisensor Integration Strategy (GMIS) and the framework for comprehensive error analysis in Discrete Kalman filtering developed through the authors’ previous research. This work enables the time-varying estimation of all individual sensor errors for an IMU array, as well as rigorous fault detection and exclusion for outlying measurements from all constituent sensors. This research explores the feasibility of applying Variance Component Estimation (VCE) to IMU array data, using separate variance components to characterize the performance of each IMU’s gyroscopes and accelerometers. This analysis is only made possible by directly modeling IMU inertial measurements under the GMIS. A real land-vehicle kinematic dataset was used to demonstrate the proposed technique. The a posteriori positioning/attitude standard deviations were compared between multi-IMU and single IMU solutions, with the multi-IMU solution providing an average accuracy improvement of ca. 14–16% in the estimated position, 30% in the estimated roll and pitch, and 40% in the estimated heading. The results of this research demonstrate that IMUs in an array do not generally exhibit homogeneous behavior, even when using the same model of tactical-grade MEMS IMU. Furthermore, VCE was used to compare the performance of three IMU sensors, which is not possible under other IMU array data fusion techniques. This research lays the groundwork for the future evaluation of IMU array sensor configurations.

## 1. Introduction

The civilian application of multi-sensor integrated kinematic positioning and navigation over the last three decades has fueled a rapid development of technologies that rely on highly accurate estimation of position, velocity, attitude, etc. In particular, the integration of GNSS and IMU sensors forms the core of modern Direct Georeferencing Technology (DGT), which assigns exterior orientations to individual image frames and/or scan lines of remote sensors without requiring the use of traditional aerial triangulation based on expensive ground control points [1,2]. DGT has seen widespread adoption, in applications ranging from aerial, land, and marine spatial data acquisition to kinematic positioning/navigation for any moving platform, including robotic and unmanned vehicles. For these applications, a high-accuracy estimate of the position and attitude of the vehicle is required, and achieving this level of accuracy can be a prohibitively expensive task.

With the development of MEMS IMUs and other low-cost positioning and orientation sensors, achieving a low-cost positioning/navigation platform while satisfying a specific high position/attitude accuracy has been a significant driver of ongoing research in academia and industry.

MEMS IMUs, while less expensive to manufacture and acquire, also tend to provide a lower accuracy than their higher-cost counterparts, with a less stable performance over time. One common method of mitigating this issue is by arranging multiple lower-cost IMUs in an array and fusing their data, simulating a higher-quality IMU than any of the constituent sensors. Practically, there are two primary ways to realize a Multiple IMU (MIMU) array:

1. Constructing an IMU array chip, such as those used in [3,4]. Array chips allow for more precise alignment of the IMU sensors in the MIMU array, and changing the direction of the constituent IMUs’ sensitive axes has an impact on the output of the MIMU array [5]. Waegli et al. [6] explored different configurations of the IMUs’ sensitive axes, finding that skew-aligned axes yield more accurate results while being more computationally challenging to integrate.
2. Constructing an IMU array from multiple strapdown IMUs that are not mounted on a common chip as described in Carlsson et al. [7]. These systems accommodate arbitrary placement of sensors, and so factors such as large lever arm vectors to individual sensors can be used to provide more precise attitude information; for example, by placing IMUs on the opposite ends of the fuselage of an airplane [5]. These systems generally have larger distances between constituent IMUs, and may not be aligned as precisely, so it is important to calibrate both lever arm vectors and boresight angles for each sensor in the array.

MIMU array processing has successfully addressed a variety of challenges faced in kinematic positioning and navigation with low-cost sensors. An MIMU array comprising IMU sensors possessing a variety of full-scale range values allows for lower-cost sensors to characterize systems that experience hugely varying system dynamics [8,9].

**Figure 1:**
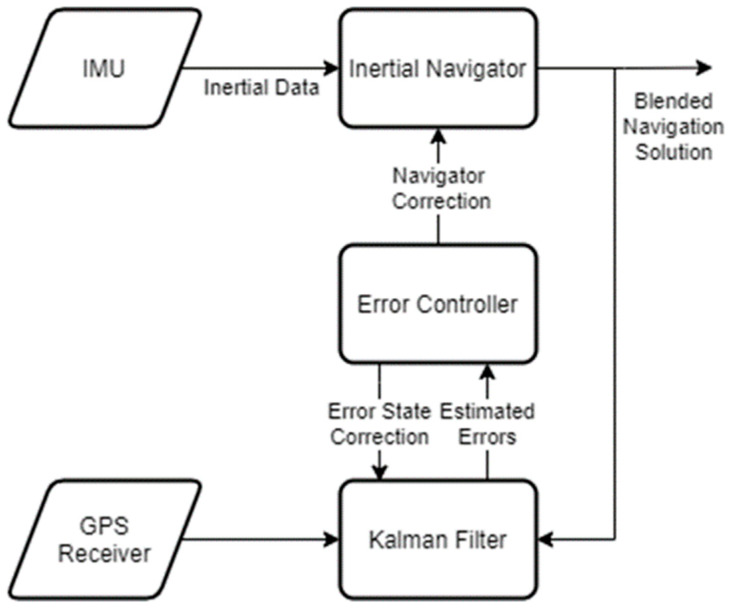
Flowchart of the traditional sensor integration strategy for a simple GPS/IMU sensor integration. This workflow was adapted from those used in [17,18,19,20].

There have been three primary methods of MIMU data fusion:

1. “Virtual” IMU [9,10,11,12,13,14]: This approach converts the MIMU output to that of a single, virtual IMU (VIMU). This generally requires transforming the data from each IMU in the MIMU array to a common reference point defining the VIMU, and this is typically accomplished through a least-squares estimation process or Kalman filter. The single VIMU’s data may then be used as an equivalent single IMU to estimate the body frame position/attitude via the traditional sensor integration strategy, whose workflow is detailed in Figure 1. The VIMU approach is very popular but suffers from a limited capacity for fault detection and exclusion, particularly for systems experiencing highly varying dynamics [12].
2. Federated Filter Fusion [11,12,15]: This approach deals with the MIMU output by dividing the overall problem into multiple single IMU positioning problems, which may then be combined with the MIMU solution via a least-squares estimation process. The Federated Filter Fusion approach also cannot accommodate fault detection and exclusion for individual IMU measurements [12].
3. Centralized Filter Fusion [11,12,16]: This approach augments the error-state estimation process to account for the MIMU output, allowing for the application of constraints on the geometrical relationships between different IMUs in the MIMU array. The Centralized Filter Fusion approach also cannot perform fault detection and exclusion for individual IMU measurements [12].

It is well known that multiple IMUs, even from the same model, often have vastly different systematic errors. In fact, they may even vary from turn-on to turn-on with the same IMU. The VIMU approach cannot accommodate individual estimates of sensor biases/scale factor errors unless this information is known *a priori* and is stable over time [12]. The Federated Filter Fusion and Centralized Filter Fusion approaches can estimate sensor biases/scale factor errors for individual IMU sensors, but sensor errors have often been assumed to be the same across individual IMUs in the MIMU array [11,12].

All three of these approaches use the traditional sensor integration strategy at their core (Figure 1). This workflow presents a consistent limitation on the potential methods to fuse MIMU array data since it only readily accommodates a single-core IMU sensor.

**Figure 2:**
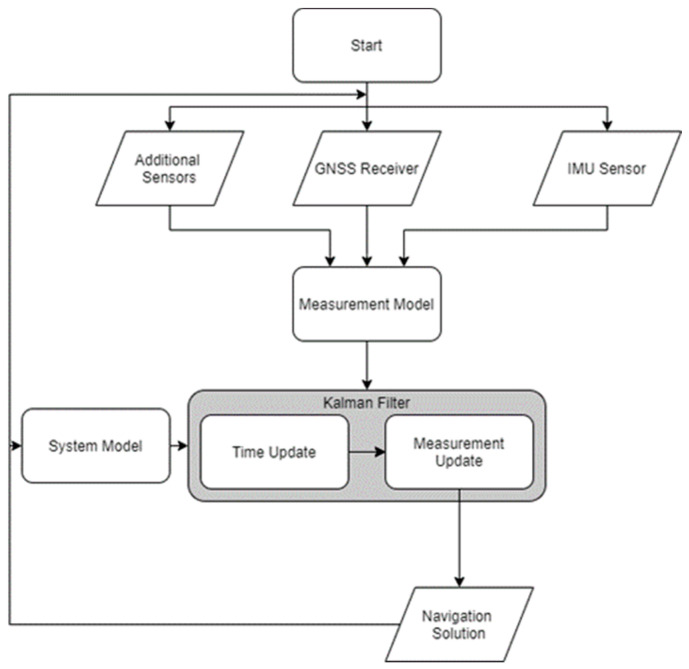
The general workflow of integrating positioning sensors in the GMIS.

One significant area of further research is in modifying the integration strategy by which position/attitude data are fused in a positioning Kalman Filter (KF) to improve the versatility/performance of MIMU array integration. The traditional multisensor integration strategy was developed to be computationally efficient, but modern developments in mobile processing power, sensors, and data science make this less of a consideration, even for real-time positioning applications. The Generic Multisensor Integration Strategy (GMIS) that has gradually been developed through [21,22,23,24,25,26] presents a valuable innovative alternative to the traditional multisensor integration at the cost of additional processing load (Figure 2). The GMIS is particularly promising in its ability to model and account for individual sensor errors for arbitrary sensor configurations, which is directly applicable to any IMU array regardless of its configuration/size. Moreover, many techniques for IMU array data fusion assume that the sensor array occupies a very small physical space such that the lever arm vectors for each IMU sensor may be assumed to be identical. The ability to individually model each IMU sensor in the array removes the need for this assumption and can accommodate sensor arrays of arbitrary size, even ones spanning the entire body frame. Additionally, since the GMIS does not rely on error state estimation, but rather directly models any positioning and angular measurements, the observation residuals for each individual IMU in an MIMU array are directly estimated, allowing for a more thorough assessment of individual time-varying IMU performance in the MIMU array. Zhu et al. [27] experimentally tested this approach using simulated GPS L1 C/A measurements and three strapdown IMUs based on the GMIS system and measurement models developed by Wang et al. [25] and Qian [28]. Our objective here is to further intensively innovate the GMIS and generalize it to MIMU arrays with arbitrary size/configuration and test it using real road test data.

By taking advantage of the GMIS, this research formulates the MIMU array integration problem with the intention of enabling a full characterization of individual IMU performance in an MIMU array in conjunction with the framework for comprehensive error analysis in discrete Kalman filtering. It includes separate IMU bias and scale error estimates for each IMU in the MIMU array, and direct estimates of each IMU’s gyroscope and accelerometer observation residuals and their variance components.

## 2. Modeling IMU Array Observations in the GMIS

### 2.1. Discrete Kalman Filtering

In general, a KF consists of a system model, which defines system kinematics, and a measurement model, which functionally relates the recorded observations to the current system states. At an arbitrary time $t_{k+1}$, these models are given as follows

(1) $$x\left(k+1\right)=A\left(k+1,k\right)x\left(k\right)+B\left(k+1,k\right)w\left(k\right)$$

and

(2) $$z\left(k+1\right)=C\left(k+1\right)x\left(k+1\right)+\Delta \left(k+1\right)$$

where $x\left(k+1\right)$ is the system state vector, A is the transition matrix that defines the system kinematic models from $t_{k}$ to $t_{k+1}$, w is the process noise vector defining the errors in the kinematic models, B is the process noise transition matrix, z is the observation vector, C is the design matrix, and Δ is the measurement noise vector.

The GMIS workflow (see Figure 2) is unique in that the measurement models used in (2) all directly model positioning observations, rather than using error measurements as the traditional integration strategy does. The elements of $z\left(k+1\right)$ therefore consist of all the raw GPS and IMU measurements at the current observation epoch.

In this research, the zero velocity/angular rate update is applied when the system is stationary. This is realized as constraint equations imposed upon the system state elements using

(3) $$H\left(x\left(k+1\right),\;k+1\right)+h\left(k+1\right)=0$$

where H is the set of constraint equations imposed upon the system state, and h is a vector of constants defined by the constraint equations.

### 2.2. Position and Attitude System Models in the GMIS

Another unique factor to the GMIS is the way that the KF system model is decoupled from the specific IMU-derived trajectory, which allows for the use of system models derived from the kinematics we expect the system to experience.

For this research, we use a constant linear acceleration model to describe the change in the system’s linear position over time, which can be expressed as

(4) $$x_{p}\left(k+1\right)=\left[\begin{array}{ccc} I_{3} & \Delta t_{k+1}I_{3} & \frac{1}{2}\Delta t_{k+1}^{2}I_{3}\\ 0_{3} & I_{3} & \Delta t_{k+1}I_{3}\\ 0_{3} & 0_{3} & I_{3} \end{array}\right]x_{p}\left(k\right)+\left[\begin{array}{c} \frac{1}{6}\Delta t_{k+1}^{3}I_{3}\\ \frac{1}{2}\Delta t_{k+1}^{2}I_{3}\\ \Delta t_{k+1}I_{3} \end{array}\right]\dot{a}\left(k\right)=A_{p}x_{p}\left(k\right)+B_{p}\dot{a}\left(k\right)$$

where $x_{p}$ is a 9 × 1 vector containing the linear position, velocity, and acceleration system states, $A_{p}$ is the transition matrix for the linear position, velocity, and acceleration system states, and $B_{p}$ is the process noise transition matrix for $\dot{a}(k)$, which is the third-order motion process noise vector, or system jerk vector, expressed in the local ENU navigation frame.

For this research, we use the roll-pitch-heading attitude model to describe the change in the system’s attitude over time. Under this attitude model, the attitude matrix from the navigation frame to the body frame is expressed as

(5) $$C_{n}^{b}=R_{2}\left(\alpha \right)R_{1}\left(\beta \right)R_{3}\left(\gamma \right)$$

where α, β, and γ are the system roll, pitch, and heading, respectively. The system angular velocity may then be expressed through the first-order time derivatives of these parameters, such that the portion of the state vector corresponding to the attitude is defined as

(6) $$x_{a}=\left[\begin{array}{cccccc} \alpha & \beta & \gamma & \dot{\alpha } & \dot{\beta } & \dot{\gamma } \end{array}\right]$$

The attitude could be modeled in different equivalent ways after the GMIS [29]. In this research, the roll-pitch-heading model is chosen, since the road test data were collected with a ground vehicle and Gimbal Lock is not expected to be a concern for these dataset, although, as can be seen in (19) in Section 2.4, 1/cosβ (β−the pitch) is not involved in principle. And the attitude angles are assumed to vary linearly with time and consider the second-order derivatives of these parameters $\left(\ddot{\alpha },\;\ddot{\beta },\;\ddot{\gamma }\right)$ to represent their associated process noise. The system model for the roll-pitch-heading attitude model is therefore defined by the set of equations

(7) $$\alpha \left(k+1\right)=\alpha \left(k\right)+\dot{\alpha }\left(k\right)\Delta t_{k+1}+\frac{1}{2}\ddot{\alpha }\left(k\right)\Delta t_{k+1}^{2}$$

(8) $$\beta \left(k+1\right)=\beta \left(k\right)+\dot{\beta }\left(k\right)\Delta t_{k+1}+\frac{1}{2}\ddot{\beta }\left(k\right)\Delta t_{k+1}^{2}$$

(9) $$\gamma \left(k+1\right)=\gamma \left(k\right)+\dot{\gamma }\left(k\right)\Delta t_{k+1}+\frac{1}{2}\ddot{\gamma }\left(k\right)\Delta t_{k+1}^{2}$$

This leads to the associated transition matrix of

(10) $$A_{a}=\left[\begin{array}{cc} I_{3} & \Delta t_{k+1}I_{3}\\ 0_{3} & I_{3} \end{array}\right]$$

and the associated process noise transition matrix of

(11) $$B_{a}=\left[\begin{array}{c} \frac{1}{2}\Delta t_{k+1}^{2}I_{3}\\ \Delta t_{k+1}I_{3} \end{array}\right]$$

### 2.3. The States in the EKF

For the navigation KF used in the GMIS, the system state vector contains elements relating to the system’s linear position, velocity, and acceleration, as well as the system’s roll-pitch-heading attitude parameters and their time derivatives. The system state vector also includes elements relating to any systematic errors that are being modeled, including the GNSS integer ambiguity parameters and IMU bias/scale factor errors. Considering that a MIMU array may contain an arbitrary number of IMU sensors, there are separate parameters defining the bias and scale factor errors for each sensor in the MIMU array. The overall state vector may therefore be partitioned into the following groups:

- Linear position, velocity, and acceleration;
- Attitude and angular velocity;
- GNSS integer ambiguity parameters; and
- IMU systematic error states for each individual IMU in the MIMU array (consisting of gyro/accelerometer bias and scale factor errors).

This partitioned state vector may be defined as

(12) $$x={\left[\begin{array}{cccccc} x_{p}^{T} & x_{a}^{T} & x_{\lambda }^{T} & x_{imu1}^{T} & \ldots & x_{imun}^{T} \end{array}\right]}^{T}$$

where the subscripts p, a, λ, and imui stand for the linear kinematic state vector (position, velocity, and acceleration vectors), the angular state vector (attitude and angular velocity vectors), the GNSS ambiguity vector, and the IMU systematic error vector for the ith IMU in the MIMU array, respectively. The corresponding transition matrix, process noise vector, and process noise transition matrix defined in (1) are, therefore, partitioned as follows:

(13) $$A=\left[\begin{array}{cccccc} A_{p} & 0 & 0 & 0 & 0 & 0\\ 0 & A_{a} & 0 & 0 & 0 & 0\\ 0 & 0 & A_{\lambda } & 0 & 0 & 0\\ 0 & 0 & 0 & A_{imu1} & \ldots & 0\\ 0 & 0 & 0 & \vdots & \ddots & \vdots \\ 0 & 0 & 0 & 0 & \cdots & A_{imun} \end{array}\right]$$

(14) $$w={\left[\begin{array}{cccccc} w_{p}^{T} & w_{a}^{T} & w_{\lambda }^{T} & w_{imu1}^{T} & \ldots & w_{imun}^{T} \end{array}\right]}^{T}$$

(15) $$B=\left[\begin{array}{cccccc} B_{p} & 0 & 0 & 0 & 0 & 0\\ 0 & B_{a} & 0 & 0 & 0 & 0\\ 0 & 0 & B_{\lambda } & 0 & 0 & 0\\ 0 & 0 & 0 & B_{imu1} & \cdots & 0\\ 0 & 0 & 0 & \vdots & \ddots & \vdots \\ 0 & 0 & 0 & 0 & \cdots & B_{imun} \end{array}\right]$$

Considering the GNSS/MIMU array integrated system used in this work, there are three types of raw measurements available:

1. Double-differenced L1 C/A and L1/L2 carrier phase observations from a pair of GPS receivers for relative positioning (one static reference and one rover);
2. Specific force observations from the three accelerometers in each strapdown IMU in the MIMU array; and
3. Angular rate observations from the three gyroscopes in each strapdown IMU in the MIMU array.

Naturally, these three sets of raw measurements can be expected to have different stochastic properties and behavior throughout a dataset, and so it is intuitive to partition the system innovation vector by observation type. Additionally, taking into account the fact that each IMU in the MIMU array can also be expected to have different stochastic properties and behavior throughout a dataset, the system innovation vector may also be partitioned to separate the measurements from each IMU sensor in the MIMU array. The system innovation vector is, therefore, partitioned as

(16) $$d={\left[\begin{array}{cccccc} d_{g1}^{T} & d_{s1}^{T} & \cdots & d_{gn}^{T} & d_{sn}^{T} & d_{G}^{T} \end{array}\right]}^{T}$$

where the subscripts gi, si, and G refer to the gyroscope observations from the ith IMU in the MIMU array, the specific force observations from the ith IMU in the MIMU array, and the GNSS observations, respectively, all observed at $t_{k+1}$.

The associated design, or Jacobian, matrix is partitioned such that the partitioned state elements are separated and the partitioned observations are also separated, as

(17) $$C=\left[\begin{array}{cccccc} C_{g1,p} & C_{g1,a} & C_{g1,\lambda } & C_{g1,imu1} & \cdots & C_{g1,imun}\\ C_{s1,p} & C_{s1,a} & C_{s1,\lambda } & C_{s1,imu1} & \cdots & C_{s1,imun}\\ \vdots & \vdots & \vdots & \vdots & \ddots & \vdots \\ C_{gn,p} & C_{gn,a} & C_{gn,\lambda } & C_{gn,imu1} & \cdots & C_{gn,imun}\\ C_{sn,p} & C_{sn,a} & C_{sn,\lambda } & C_{sn,imu1} & \cdots & C_{sn,imun}\\ C_{G,p} & C_{G,a} & C_{G,\lambda } & C_{G,imu1} & \cdots & C_{G,imun} \end{array}\right]$$

This partitioning simplifies the process of modeling the individual IMU sensors in the MIMU array, and clearly documents their impact on the partitioned elements of the state vector.

It is important to note that many elements of this partitioned design matrix will be null matrices. The GNSS observations are modeled independently of any IMU systematic error parameters, so $C_{G,imui}=0\forall i\in 1,\;\ldots ,\;n$. The gyro and specific force observations are not dependent on the GNSS ambiguity parameters, so $C_{gi,\lambda }=0,C_{si,\lambda }=0\forall i\in 1,\;\ldots ,\;n$. Lastly, the observations from any IMU are only considered to be related to the systematic error parameters for that IMU, so $C_{gi,imuj}=0,C_{si,imuj}=0\forall i\neq j,\;i\in 1,\;\ldots ,\;n,\;j\in 1,\;\ldots ,\;n$.

### 2.4. IMU Observation Equations in the GMIS

In general, the gyroscope observations for the ith IMU sensor in the MIMU array may be modeled as

(18) $${R_{i}\left(\omega_{ib}^{b}\right)}_{i}=\omega_{nb}^{b}-b_{gi}-S_{gi}\omega_{ib,meas}^{b}+C_{n}^{b}\left(\omega_{ie}^{n}+\omega_{en}^{n}\right)+\Delta_{\omega_{ib}^{b}}$$

where $\omega_{ib}^{b}$ denotes the gyro measurement vector of the body frame’s angular rate with respect to the inertial frame, observed in the body frame, $b_{gi}$ denotes the gyroscope bias vector, $S_{gi}$ denotes the 3 × 3 gyroscope scale factor error matrix, $\omega_{ie}^{n}$ is the angular velocity vector of Earth with respect to the inertial frame, expressed in the navigation frame, $\omega_{en}^{n}$ denotes the angular velocity vector of the navigation frame with respect to Earth in the navigation frame, $\Delta_{\omega_{ib}^{b}}$ is the observation noise vector for the gyroscope measurements, and $R_{i}$ is the rotation matrix to align the ith IMU sensor in the MIMU array to the right-forward-up orientation with respect to the body frame. It is worth noting the importance of ensuring that the IMU sensors are properly calibrated to have their outputs aligned before integrating the data from the MIMU since boresight calibration is not accounted for in the set of IMU systematic errors in the state vector.

After Salychev [30], the differential equation relating $\omega_{nb}^{b}$ and $\left(\dot{\alpha },\;\dot{\beta },\;\dot{\gamma }\right)$ is given as

(19) $$\omega_{nb}^{b}=\left[\begin{array}{ccc} 0 & \cos\alpha & \sin\alpha \cos\beta \\ 1 & -\sin\beta & 0\\ 0 & \sin\alpha & -\cos\alpha \cos\beta \end{array}\right]\left[\begin{array}{c} \dot{\alpha }\\ \dot{\beta }\\ \dot{\gamma } \end{array}\right]$$

Substituting (19) for (18) results in the following definition of the non-zero design matrix submatrices for the gyro observations of the ith IMU:

(20) $$C_{gi,a}=\left[\begin{array}{cc} 0_{3} & \begin{array}{ccc} 0 & \cos\alpha & \sin\alpha \cos\beta \\ 1 & -\sin\beta & 0\\ 0 & \sin\alpha & -\cos\alpha \cos\beta \end{array} \end{array}\right]$$

(21) $$C_{gi,imui}=\left[\begin{array}{cccc} I_{3} & \begin{array}{ccc} \omega_{ib,measx}^{b} & 0 & 0\\ 0 & \omega_{ib,measy}^{b} & 0\\ 0 & 0 & \omega_{ib,measz}^{b} \end{array} & 0_{3} & 0_{3} \end{array}\right]$$

The accelerometer observations for the ith IMU sensor in the MIMU array may be modeled as

(22) $$R_{i}{\left(f_{ib}^{b}\right)}_{i}=C_{n}^{b}a+C_{n}^{b}g-b_{ai}-S_{ai}f_{ib}^{b}+C_{n}^{b}\left\{2\omega_{ie}^{n}+\omega_{en}^{n}\right\}\times v+C_{n}^{b}\omega_{ib}^{b}\times \omega_{ib}^{b}\times r_{i}+\Delta_{f_{ib}^{b}}$$

where $f_{ib}^{b}$ denotes the specific force the body frame experiences with respect to the inertial frame, expressed in the body frame, a denotes the acceleration of the body frame in the navigation frame at $t_{k+1}$, g denotes the local gravity vector, expressed in the navigation frame; v denotes the velocity of the body frame in the navigation frame, r denotes the lever arm vector for the IMU sensor in the body frame, $\Delta_{f_{ib}^{b}}$ is the observation noise vector for the accelerometer measurements, and $R_{i}$ is the rotation matrix to align the ith IMU sensor in the MIMU array to the right-forward-up orientation with respect to the body frame.

Taking the partial derivatives of (22) with respect to the elements of the state vector results in the following definition of the non-zero design matrix submatrices for the accelerometer observations on the ith IMU:

(23) $$C_{si,p}=\left[\begin{array}{ccc} 0_{3} & 0_{3} & C_{n}^{b} \end{array}\right]$$

(24) $$C_{si,a}=\left[\frac{\partial C_{n}^{b}}{\partial x_{a}}\left(a+g+\left\{2\omega_{ie}^{n}+\omega_{en}^{n}\right\}\times v+\omega_{ib}^{b}\times \omega_{ib}^{b}\times r_{i}\right)\right]$$

(25) $$C_{si,imui}=\left[\begin{array}{cccc} 0_{3} & 0_{3} & I_{3} & \begin{array}{ccc} f_{ib,measx}^{b} & 0 & 0\\ 0 & f_{ib,measy}^{b} & 0\\ 0 & 0 & f_{ib,measz}^{b} \end{array} \end{array}\right]$$

## 3. Error Analysis

The sources of error in a Discrete KF may generally be separated into three statistically independent components:

1. Measurement Noise: This noise is indicative of errors in individual positioning/angular observations, or their model equations. This noise vector may be partitioned to separately consider each individual positioning/attitude sensor.
2. Process Noise: This noise is indicative of errors in the system kinematic models and may be used to characterize the varying dynamic performance of the system.
3. System State Noise: This noise is indicative of errors in the system state estimation process and is affected by accumulated errors from previous epochs.

The three associated residual vectors may be calculated via the approach taken in Wang [21] for the positioning EKF used in this research. When the system is stationary and the zero velocity/angular rate update is being applied via constraint equations, the system state-constrained KF framework is applied to estimate the process noise, system state, and observation residuals [31]:

(26) $$v_{h}^{x}=v^{x}-AD_{xx}A^{T}{\left(I-GC\right)}^{T}H^{T}{\left(HD_{xx}H^{T}\right)}^{-1}h$$

(27) $$v_{h}^{w}=v^{w}-QB^{T}{\left(I-GC\right)}^{T}H^{T}{\left(HD_{xx}H^{T}\right)}^{-1}h$$

(28) $$v_{h}^{z}=v^{z}-CD_{xx}H^{T}{\left(HD_{xx}H^{T}\right)}^{-1}h$$

where $v_{h}^{x}$, $v_{h}^{w}$, and $v_{h}^{z}$ are the constrained residual vectors corresponding to the system state elements, process noise, and observation vectors, respectively; G is the Kalman Gain matrix; Q is the covariance matrix of the process noise vector; and h is the misclosure vector for the constraint equations that are being applied. $v^{x}$, $v^{w}$, and $v^{z}$ are the associated residual vectors calculated via [21].

When focusing on comparing the performance of individual sensors in an MIMU array, the observation residual vector is of primary concern. The ability to directly estimate observation residuals for the raw IMU gyroscope and accelerometer measurements is unique to the GMIS, and these quantities were notably used to perform Variance Component Estimation (VCE) in [29]. This research is also well suited to leveraging VCE to characterize IMU performance. This involves normalizing the quadratic form $v_{i}^{T}D_{v_{i}v_{i}}^{-1}v_{i}$ for a subset of residual values by their degrees of freedom (DOF), or contribution to the overall system redundancy. The DOF contribution of any subset of a residual vector may be calculated as follows [23,32]:

(29) $$DOF_{i}=tr\left(D_{v_{i}v_{i}}D_{l_{i}l_{i}}^{-1}\right)$$

where $D_{v_{i}v_{i}}$ denotes the covariance matrix for the residual vector $v_{i}$ being used for VCE and $D_{l_{i}l_{i}}$ denotes the a priori covariance matrix for the corresponding observation vector $l_{i}$.

When conducting VCE, it is important to bear the application in mind so that any subsets of the residual vectors that are being used provide the most meaningful information about what is being studied. In this paper, research is primarily focused on characterizing individual IMU performance in an MIMU array, so the following sets of variance components are calculated:

For the measurement residuals:

1. An overall variance component describing the performance of the entire observation vector. This provides an overview of where there may be issues in the observations, regardless of their specific source.
2. Separate variance components dedicated to characterizing the performance of the raw gyroscope and accelerometer measurements for each IMU in the MIMU array. This provides overall performance measures for each individual IMU that may be directly compared across different IMUs to compare their performance over the observation period.
3. Variance components dedicated to characterizing the performance of the GPS double-differenced observations, including one for the L1 C/A, L1 Carrier Phase, and L2 Carrier Phase observations.

For the process noise residuals, an overall variance component describes the performance of the entire process noise vector. This provides information about the overall performance of the kinematic models used over the dataset.

It is important to clarify at this point that the overall DOF for a KF is equal to the number of recorded observations plus the number of system state constraints at a given point in time and that the sum of the DOF values for *all three residual vectors* will be equal to the overall DOF for the KF. Practically speaking, this means that some elements of the residual vectors have very small contributions to the overall system redundancy at any observation epoch and may, therefore, produce unreliable variance component estimates when calculated on an epoch-by-epoch basis. As a result, variance components are rather estimated over a period of time using the cumulative sum of both the residual subsets’ quadratic forms and the corresponding DOF values.

It is also worth noting that, since VCE is performed using the estimated residuals for the observation, process noise, and system state vectors, the performance information characterized by the variance components may be estimated without requiring a ground truth or reference solution. This means that a detailed analysis of a positioning system’s stochastic properties may be performed even in the absence of a reference solution.

## 4. Land Vehicle Test Data and Analysis of Results

### 4.1. Definition of the Road Test Dataset

This section presents the results from a real kinematic dataset collected using our in-house integrated navigation system consisting of three VectorNav VN-100 IMUs (operating at 100 Hz) and a NovAtel OEM6 receiver (operating at 100 Hz) mounted on a land vehicle while a second NovAtel OEM6 receiver was operating at a separate fixed location as a base station.

**Figure 3:**
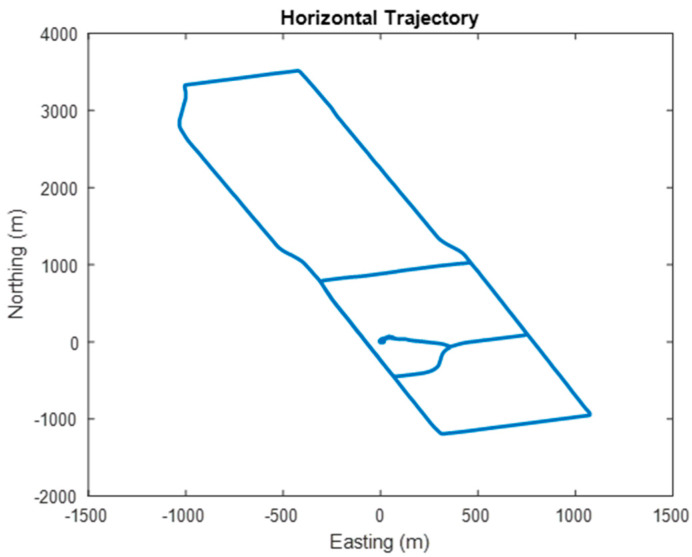
The top-down view of the trajectory of the kinematic dataset. The coordinates presented are local geodetic coordinates relative to the starting location.

**Figure 4:**
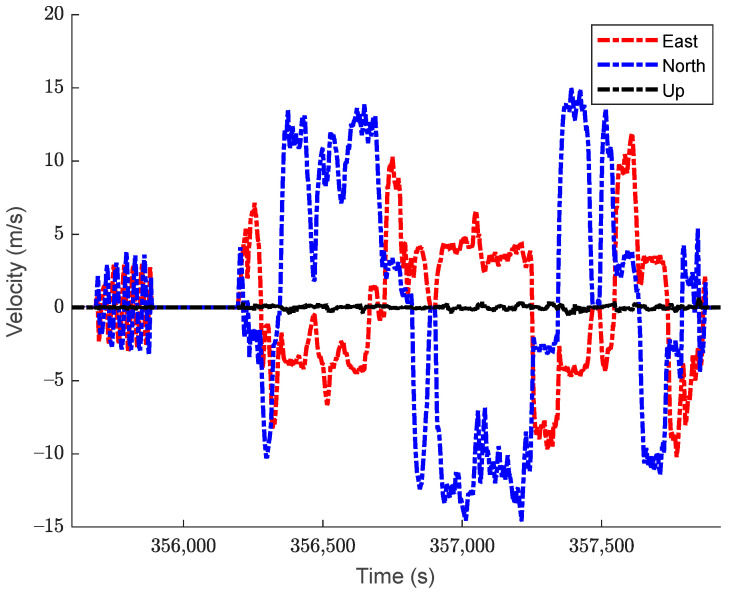
The velocity profile of the kinematic dataset. Velocity values are expressed in the navigation frame of local geodetic coordinates.

**Figure 5:**
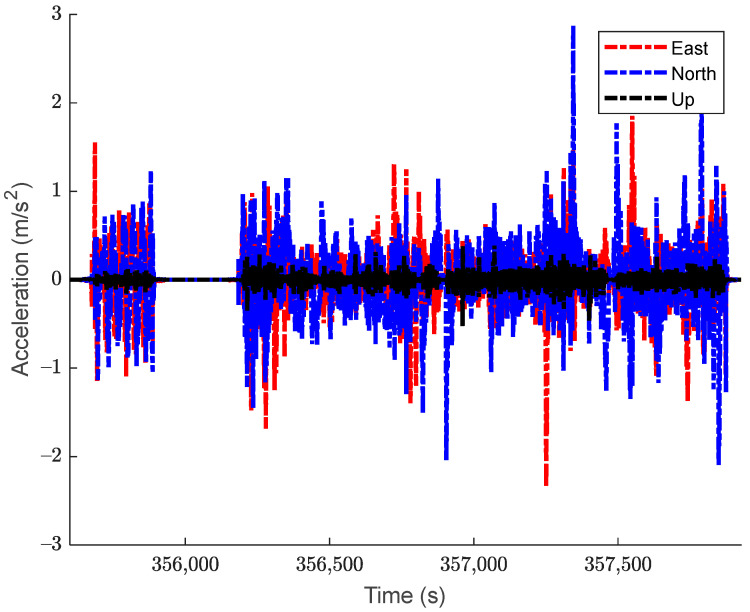
The acceleration profile of the kinematic dataset. Acceleration values are expressed in the navigation frame of local geodetic coordinates.

**Figure 6:**
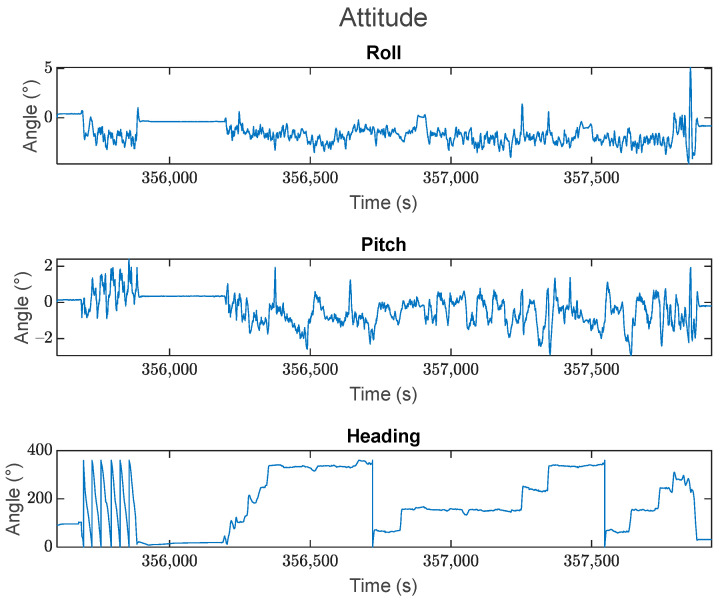
The roll, pitch, and heading profiles for the kinematic dataset. Attitude is presented in the local navigation frame.

**Figure 7:**
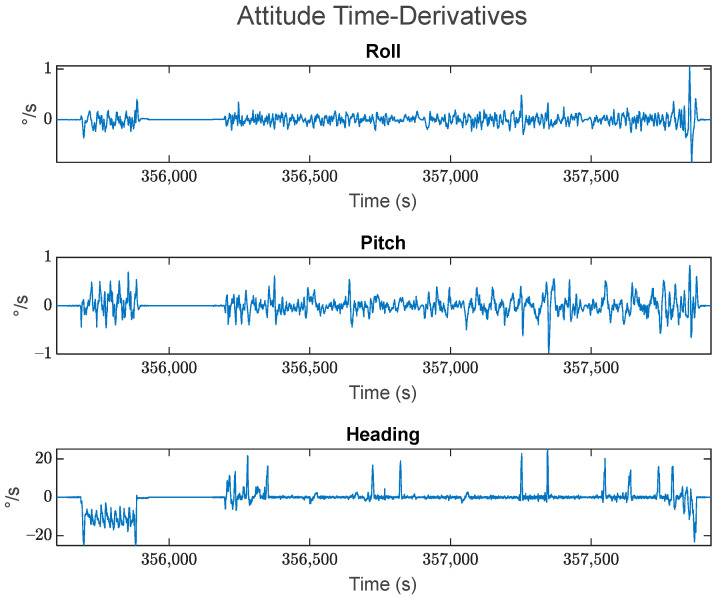
The estimated time derivatives for the roll, pitch, and heading values over the kinematic dataset. These values are presented in the local navigation frame.

The test dataset is approximately 50 min long. The vehicle remained stationary for the first 5 min, after which it spent about 5 min moving in circles around a residential court, then it spent another 5 min in stationary. After this, the vehicle was then driven in kinematic mode for approximately 30 min, ending with another 5 min in stationary. The top-down view of the trajectory is shown in Figure 3, with the corresponding velocity and acceleration profiles displayed in Figure 4 and Figure 5, respectively. The estimated roll-pitch-heading profiles, as well as their estimated time derivatives, are shown in Figure 6 and Figure 7, respectively.

It is worth noting the sudden jump in the estimated system roll in Figure 6 towards the end of the dataset. This corresponds to the vehicle going over a curb as it pulled into a driveway immediately before the final stationary period in the dataset.

After the GMIS, the data from all three constituent IMUs were able to be directly integrated into the EKF measurement update. This research focuses on analysis tasks that are typically not possible to achieve under either the traditional integration strategy or other MIMU array data fusion strategies, namely:

1. Direct individual estimation of any systematic errors each sensor in the MIMU array is experiencing, without any assumptions regarding consistency in systematic errors between different IMU sensors. This is explored in Section 4.2.
2. Direct estimation of the measurement residuals for each IMU sensor in the MIMU array. Additionally applying VCE allows for a direct comparison between the overall performance of different sensors in the MIMU array. This is explored in Section 4.3.

### 4.2. Estimated Sensor Systematic Errors and Measurement Residuals

**Figure 8:**
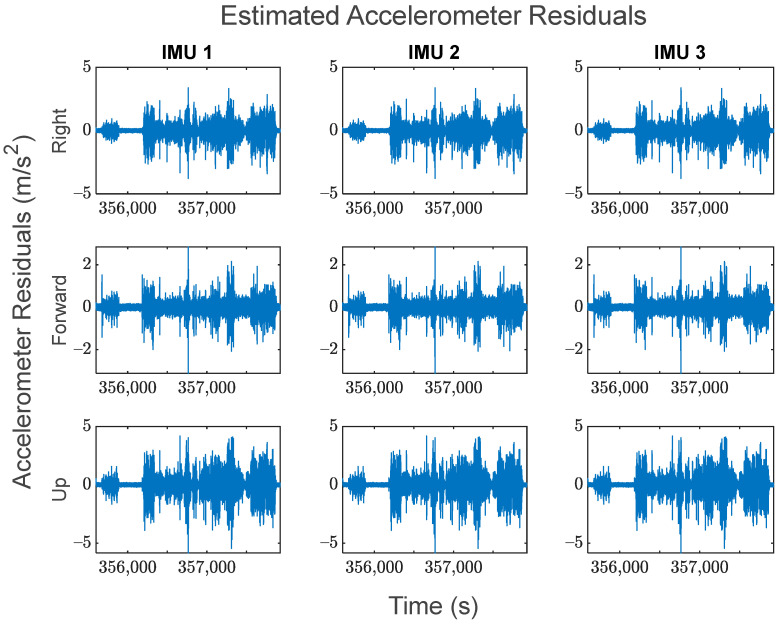
The estimated accelerometer-specific force residuals of each IMU in the array for the kinematic dataset.

**Figure 9:**
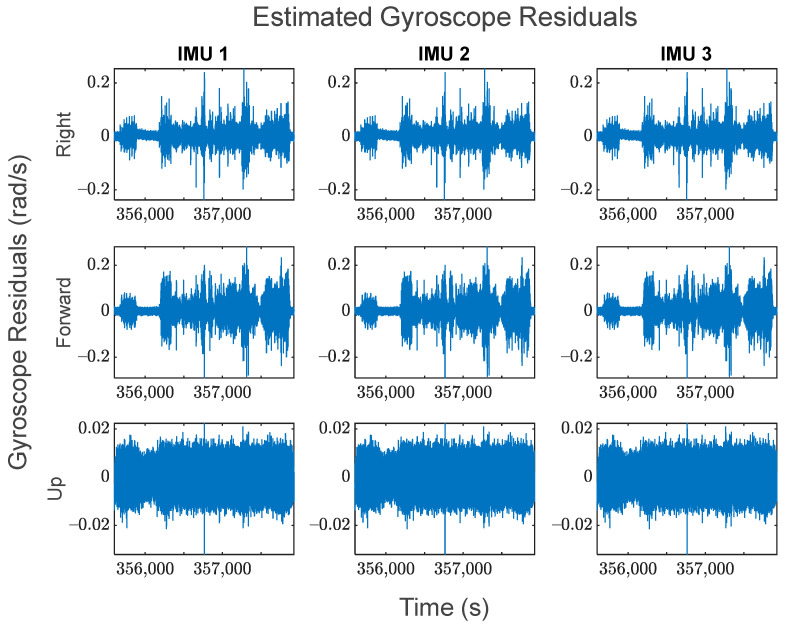
The estimated gyroscope angular rate residuals of each IMU in the array for the kinematic dataset.

**Figure 10:**
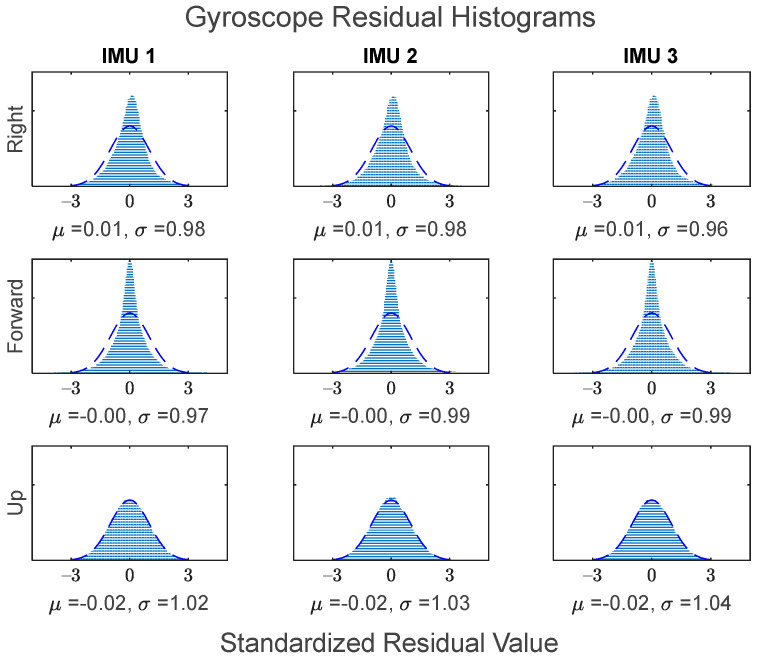
Histograms of the gyroscope standardized residuals for all three constituent IMUs. Standard normal distribution superimposed for reference.

**Figure 11:**
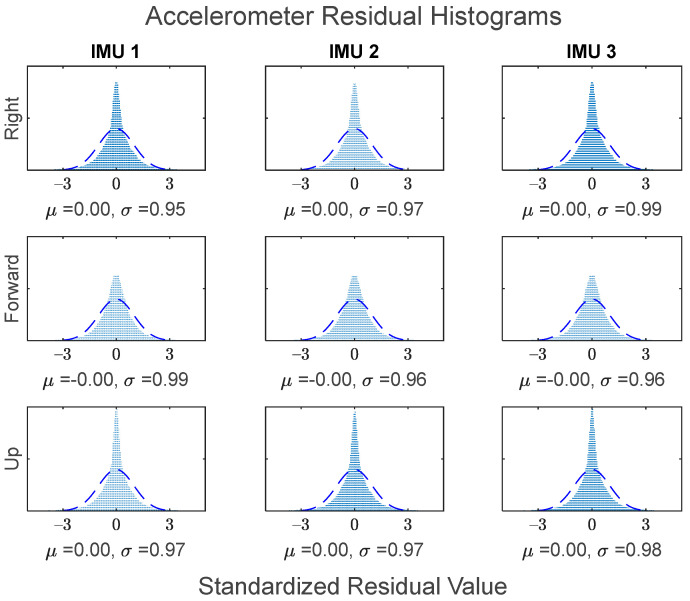
Histograms of the accelerometer standardized residuals for all three constituent IMUs. Standard normal distribution superimposed for reference.

The GMIS is unique in its ability to directly model raw IMU measurements, and to therefore estimate their residuals after the EKF measurement update beyond the widely used system innovations. Since IMUs are modeled individually as part of the GMIS’ EKF, separate sets of the residuals of the raw IMU measurements are estimated for all sensors comprising the MIMU array. The accelerometer-specific force residuals for the kinematic dataset are shown in Figure 8, and the gyroscope angular rate residuals for the kinematic dataset are shown in Figure 9. Histograms for all estimated IMU observation residuals are shown in Figure 10 and Figure 11. The analysis in this research is focused on the IMU array data, and so the analysis related to the GPS measurements is not presented as much in this manuscript.

There are two notable patterns exhibited by these raw IMU residuals:

1. The behavior of the IMU measurement residuals is very consistent across the three constituent IMU sensors in the MIMU array. Since all three of these sensors are of the same model, this suggests that all IMU measurements have been integrated successfully through the GMIS.
2. The behavior of the IMU measurement residuals is highly dependent upon the time-varying dynamics that the system is experiencing. From Figure 8 and Figure 9, it is very clear that each sensor experiences much larger random noise while the system is in motion, and the times of highest random noise occur when a maneuver is being undertaken.

These residuals may be used to generate histograms to further characterize the overall behavior of the observations over the entirety of the dataset. Figure 10 shows the standardized residual histograms for the gyroscope observations for each IMU in the MIMU array, and Figure 11 shows the standardized residual histograms for the accelerometer observations for each IMU in the MIMU array.

The IMU measurement residuals in Figure 10 and Figure 11 were quite well behaved overall. Many of these histograms have sharp peaks near zero, which is due to the fact that the constituent IMUs generally experienced less noise when the vehicle was stationary.

**Figure 12:**
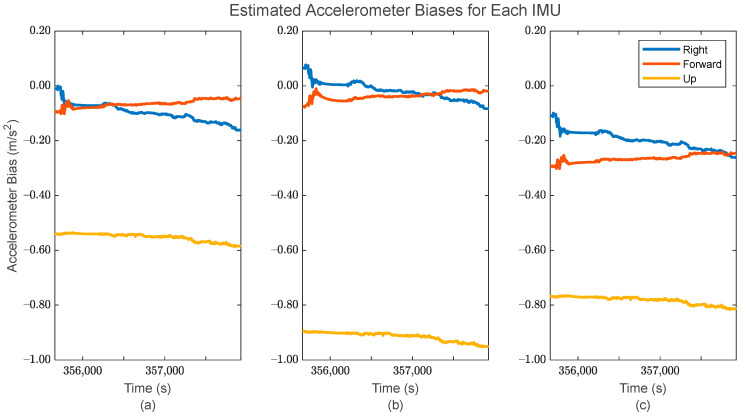
The estimated accelerometer bias for (**a**) the first IMU in the array, (**b**) the second IMU in the array, and (**c**) the third IMU in the array.

**Figure 13:**
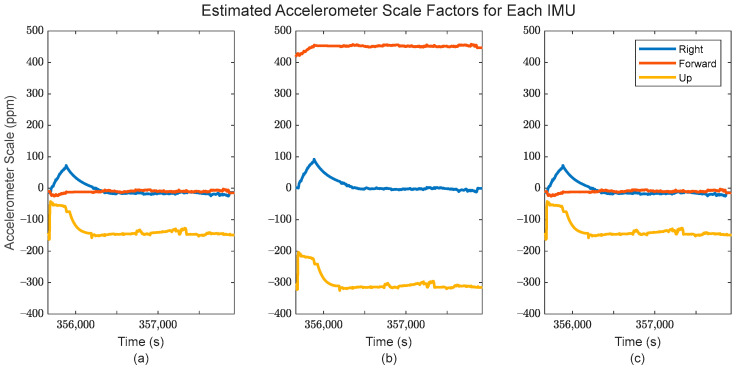
The estimated accelerometer scale factor errors for (**a**) the first IMU in the array, (**b**) the second IMU in the array, and (**c**) the third IMU in the array.

**Figure 14:**
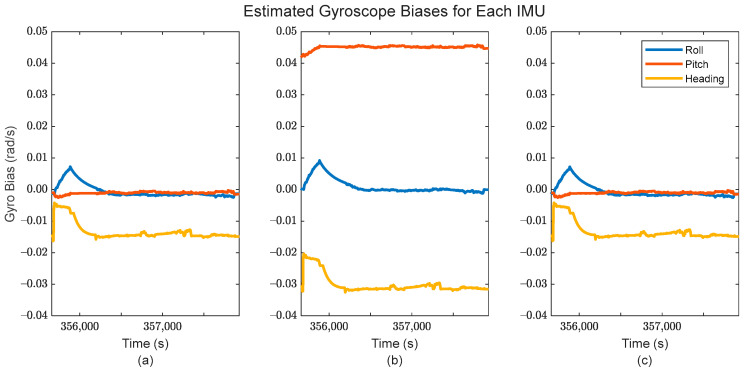
The estimated gyroscope bias for (**a**) the first IMU in the array, (**b**) the second IMU in the array, and (**c**) the third IMU in the array.

**Figure 15:**
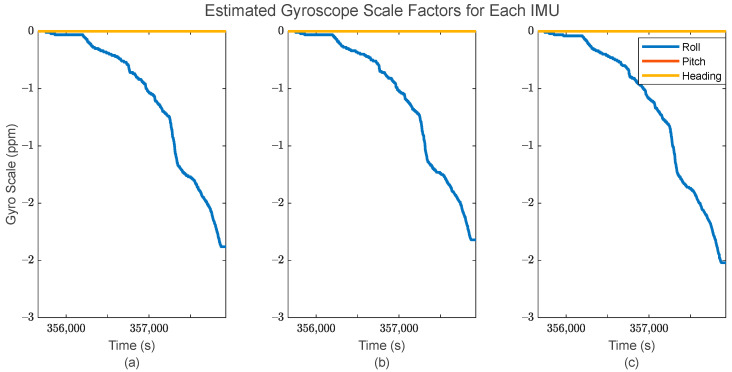
The estimated gyroscope scale factor errors for (**a**) the first IMU in the array, (**b**) the second IMU in the array, and (**c**) the third IMU in the array.

Additionally, the application of IMU array processing after the GMIS allows for individualized sensor systematic error estimation, i.e., estimation of the biases/scale factor errors associated with the accelerometers and gyroscopes. These estimates are shown in Figure 12 and Figure 13 for the accelerometers, and Figure 14 and Figure 15 for the gyroscopes.

In Figure 12 and Figure 14, it is clear that the estimated individual IMU biases remain relatively consistent over time for the constituent IMUs, which is to be expected when using tactical-grade IMUs. The bias estimates for each sensor are also clearly different, which suggests that the formulation of the EKF in the GMIS has properly accounted for the different sensor biases of each constituent IMU.

From Figure 13 and Figure 15, it is clear that the estimated individual IMU scale factor errors are reasonably consistent throughout the dataset. It is worth noting that the scale factor error estimates are generally much larger for the accelerometers than for the gyroscopes. This is likely due to the accelerometers having a larger numerical range of values over the dataset than the gyroscopes. As with the bias estimates, the estimated scale factor errors are generally different for different sensors, suggesting that they should be modeled individually per each IMU as here in the EKF after the GMIS, instead of using a group of shared states among multiple IMUs.

### 4.3. Characterizing Sensor Performance with VCE

The ability to estimate the residuals of the raw measurements of accelerometers and gyroscopes additionally allows VCE to characterize the performance of different parts of the EKF with the aid of their measurement redundancy indexes [31]. The variance components associated with the observations provide a time-varying overview of how well the sensor observations fit their observation models on the whole. In practice, the variance factors or components can be estimated at multiple levels for different purposes. Two of the most essential variance components are provided in this section: the standard observation variance factor of unit weight and the variance components of the individual measurement types.

**Figure 16:**
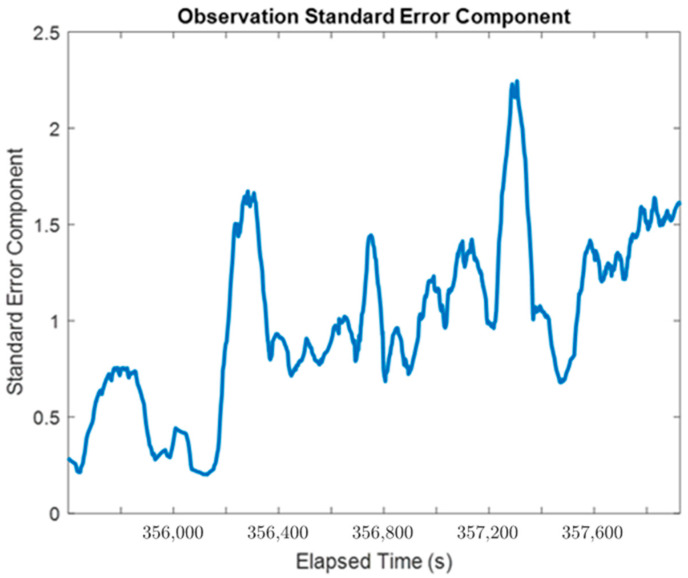
The estimated overall standard error of unit weight for the MIMU-integrated system (moving window: 20 s).

First, the overall standard error of unit weight for the entire EKF as a Least-Squares system is shown in Figure 16, calculated over 20 s intervals. It is clear that the overall quality of the observations was generally higher when the system was experiencing less dynamic movement (i.e., better performance during the two stationary periods in the dataset and during the relatively slow movement of the vehicle around the residential court) as it reflects not only the errors in measurements and also the process noises in the system model. For the majority of the rest of the kinematic dataset, standard error components hovered at around a value of 1, which suggests that the overall performance of the positioning sensors is in line with their presumed a priori accuracies.

**Figure 17:**
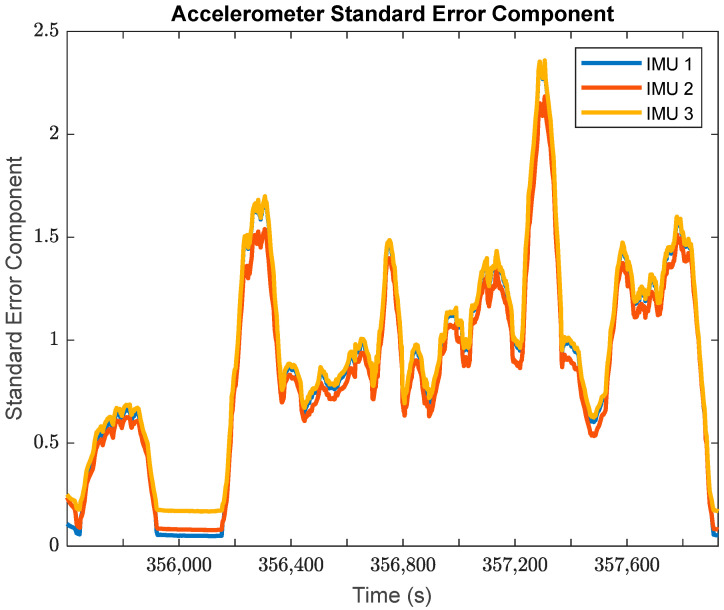
The estimated standard errors of unit weight for the specific force measurements of three sets of IMU accelerometers in the MIMU-integrated system (moving window: 20 s).

**Figure 18:**
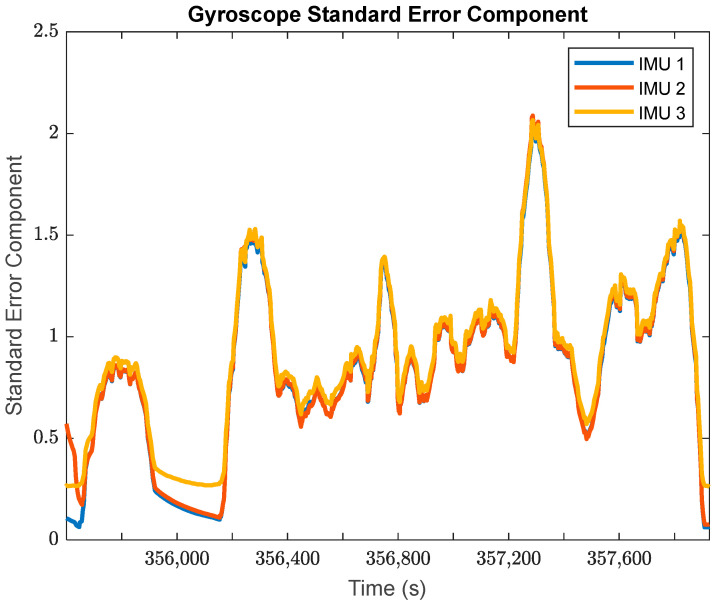
The estimated standard errors of unit weight for the angular rate measurements of three sets of IMU gyroscopes in the MIMU-integrated system (moving window: 20 s).

Furthermore, looking more granularly and separating the constituent parts of the overall observation standard errors of unit weight per measurement type provides the estimated standard error components in Figure 17 and Figure 18.

Figure 17 and Figure 18 provide further evidence to support that the IMU performance varies with the system dynamics. In both of these plots, each IMU is characterized by low standard error components, and therefore better performance for the stationary periods in the kinematic dataset. Furthermore, both the estimated gyroscope and accelerometer standard error components spike over the periods of time when the land vehicle was turning on the road.

It is worth noting that the three IMUs exhibited very similar performance over the course of the kinematic dataset, with nearly identical standard error components over the entire period. Their similar performance is encouraging, since they are all the same model of IMU, and it is instructive to see that their performance is so similar as they should exercise the same level of random noises while their systematic error estimates are quite different from one another.

The global estimates of the standard error components in Figure 17 and Figure 18 may be used to estimate the a posteriori observation noise for each observation type in the dataset. These a posteriori estimates are summarized in Table 1 for the IMU observations.

### 4.4. Comparing MIMU Array Results to Single-IMU Results

**Figure 19:**
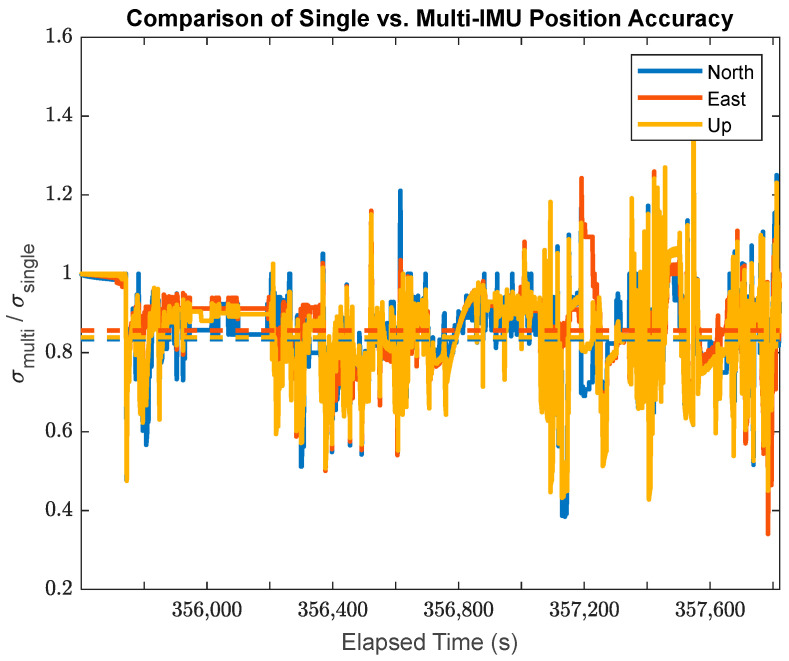
The ratio of the a posteriori MIMU position standard deviations to the a posteriori SIMU position standard deviations (Note: the dashed lines plot the average ratios for each sensor axis).

**Figure 20:**
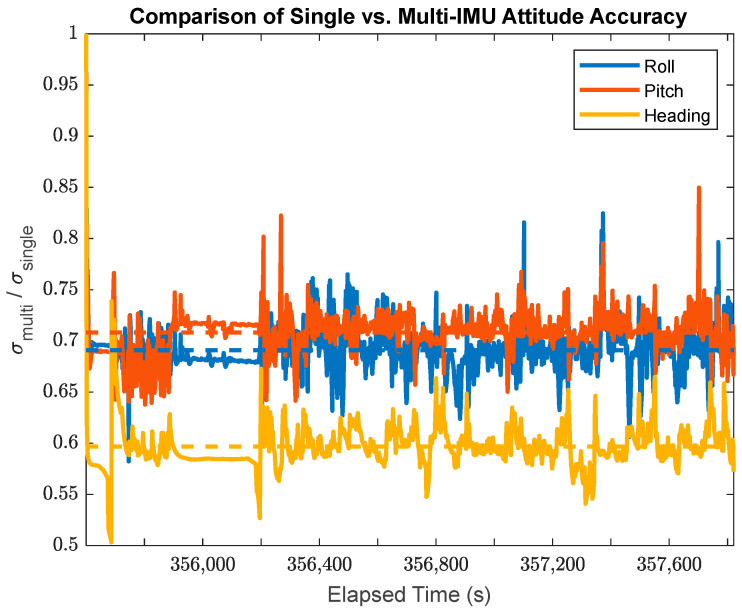
The ratio of the a posteriori MIMU attitude standard deviations to the a posteriori SIMU attitude standard deviations (Note: the dashed lines plot the average ratios for each sensor axis).

**Figure 21:**
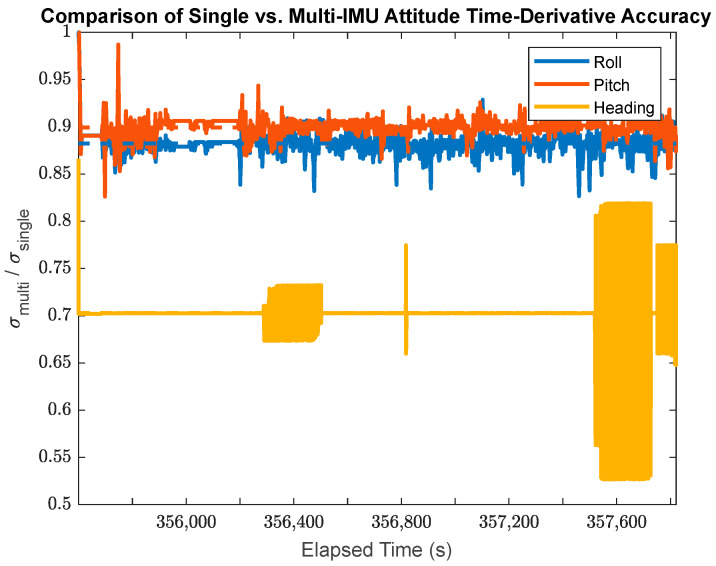
The ratio of the a posteriori MIMU attitude time-derivative standard deviations to the a posteriori SIMU attitude time-derivative standard deviations (Note: the dashed lines plot the average ratios for each sensor axis).

This research also facilitates a direct comparison between the single-IMU (SIMU) positioning/orientation results and the MIMU array positioning results. The time-varying a posteriori standard deviations for the SIMU and MIMU may be directly compared using their ratio at any given point in time as shown in Figure 19 for the position accuracy, in Figure 20 for the attitude accuracy, and in Figure 21 for the accuracy of the attitude time-derivatives.

In Figure 19, the data from the MIMU array system presents an average position accuracy improvement of 16.6% in the North component, 14.4% in the East component, and 16.1% in the Up component over the SIMU solution. This makes it clear that there is an overall improvement in positioning quality when using multiple IMUs as an MIMU array. Looking at the ratios of the time-varying standard deviations, this improvement is much more pronounced when the system is experiencing higher dynamics, for example, during a turn (a good example of this occurs at approximately t = 357,250 s). Overall, the position accuracy is less impacted by the availability of MIMU data than the attitude accuracy, due to the fact that the position accuracy primarily comes from the available GPS measurements.

In Figure 20, the data from the MIMU array system show an average accuracy improvement in attitude of 29.6% in roll, 31.1% in pitch, and 40.2% in the heading over the SIMU solution. This shows the most dramatic improvement of any of Figure 19, Figure 20 and Figure 21. There are several factors that feed into the general performance of the MIMU attitude solution:

1. Since all constituent IMU sensors in the MIMU array are assumed to conform to rigid body motion, the ongoing estimates of individual sensor biases are much more reliable than they would be for the SIMU solution, which allows for the data from the individual IMU sensors to be more reliable than they otherwise would be. It is important to note that under situations where the sensors were not static relative to each other (e.g., on the fuselage of an airplane), the dynamics of that motion would also need to be accounted for.
2. The accuracy improvement of roll and pitch angles is much less pronounced than the accuracy improvement of the heading. This is due to the fact that roll and pitch can generally be estimated with relatively high accuracy from IMU accelerometer data. This is commonly performed as part of the coarse leveling process, but the accelerometers still provide this level of attitude information throughout a kinematic dataset, even when using tactical grade MEMS IMUs.
3. Here, the data collection system was land vehicle-based, without highly varying dynamics in the pitch and roll angles in general. The time-varying improvements in attitude accuracy remain much more consistent over time than for the position solution, varying much less over the course of the entire dataset.

In Figure 21, the MIMU array data shows an average attitude time-derivative accuracy improvement over the SIMU solution of 11.7% in the estimated pitch, 10.1% in the estimated roll, and 29.7% in the estimated heading. As with the attitude parameter performance, the accuracy improvement in the roll and pitch time derivatives is much less pronounced than the accuracy improvement for the heading time derivatives. Again, this is due to the fact that the IMU accelerometers provide accurate information about the system roll and pitch, which effectively also constrains the estimates of their time derivatives.

**Figure 22:**
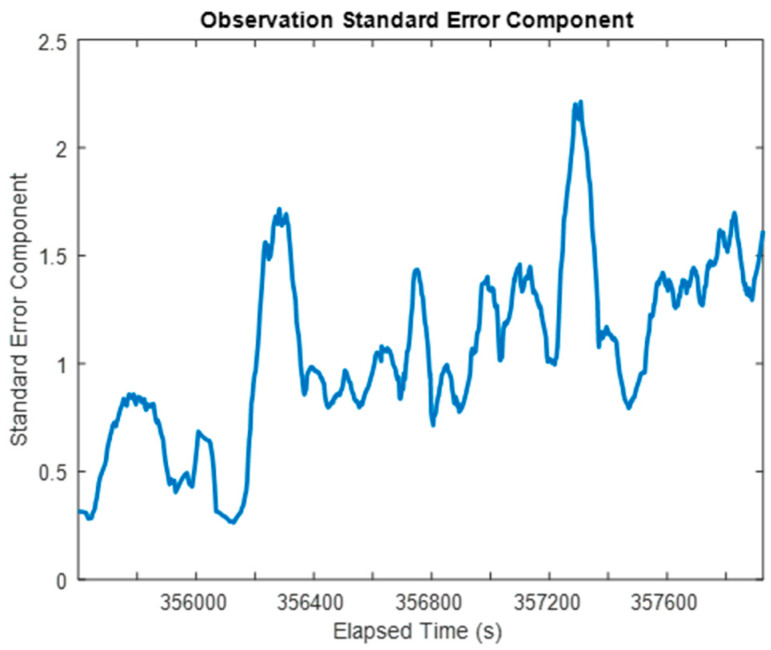
The estimated standard errors of unit weight for the SIMU-integrated system (moving window: 20 s).

It is also instructive to compare the estimated SIMU standard error components with those of the MIMU array solution. Figure 22 shows the overall standard errors of unit weight for the whole observation vector, which may be compared with the MIMU array overall standard errors of unit weight in Figure 16. As expected, these two plots are very similar to one another, reflecting that the overall system performance relative to the a priori assumed observation accuracies is consistent across both the MIMU and SIMU solutions.

## 5. Conclusions

This research proposed a distinct innovative modeling technique that can estimate the individual time-varying systematic errors of each IMU when applied to an MIMU array integrated positioning and orientation system by taking advantage of the GMIS data fusion strategy. Moreover, this research’s inclusion and expansion of the KF error analysis framework from [31] allows for the proposed technique to be used in a comprehensive error analysis that would not otherwise be possible for MIMU array data. The outcomes of this research confirmed its success and showed its unique advantages over the other existing techniques for modeling IMU array integrated systems. In the traditional multisensor integration strategy, IMU systematic errors are commonly treated as homogeneous across all the IMUs as one must have an IMU as the core sensor to generate the error states and error measurements. This proposed technique instead incorporates all individual IMU bias and scale factor errors in the system state vector and utilizes the individual IMU data directly through the measurement update in the EKF. This allows for much more detailed information to be gleaned about the behavior of the IMU systematic errors and also allows more granular information about the performance of individual IMU sensors in the MIMU array.

As can be seen from the estimated systematic errors presented in Section 4.2, one cannot simply assume that the systematic errors for individual IMU sensors exhibit homogeneous behavior, and it is therefore important to model individual sensors’ systematic errors for rigorous position/orientation estimation. Additionally, with the innovative processing framework of the GMIS, it is readily possible to change the systematic error models for IMU sensors in the MIMU array integrated system, for example, by estimating cross-coupling errors, sensor lever arm vectors, and/or sensor bore-sight angles in the EKF. This novel multi-sensor integration strategy allows for the testing of multiple systematic error models or stochastic models to provide in-depth information about how expanding the systematic error models affects the position/orientation solution. Additionally, using Variance Component Estimation to characterize individual sensor performance provides new avenues for online evaluation of IMU data quality for fault detection and exclusion.

The models put forth for this research were fully realized and successfully tested on real road data, providing powerful tools for characterizing the performance of the positioning EKF, particularly when analyzing the performance of each IMU sensor.

This approach to IMU array processing is readily extendable to different arbitrary sensor configurations, which would allow for a more detailed analysis of different hardware configurations and the impact they have on positioning solution quality. The characterization of individual IMU sensor errors makes this approach well suited to identifying how factors such as IMU spacing and orientation impact the quality of the positioning solution, and this is an important future research topic.

There are some limitations to using ground vehicle data, namely that the system’s roll and pitch experience lower dynamics. While these data are valuable in validating the models developed for this research, it will be valuable in the future to test the performance improvement of the MIMU array solution over the SIMU solution for airborne data to gain a better understanding of the roll and pitch estimates’ improvement when their values experience more dynamic changes.

## Figures and Tables

**Table 1:** Overall estimated a posteriori standard deviations for each IMU observation type. These values were estimated by scaling the a priori standard deviation estimate by the global estimate of the standard error of unit weight. Values were estimated separately for each IMU in the MIMU array.

	A Posteriori Standard Deviations	
IMU Sensor Number	Accelerometer [m/s^2^]	Gyroscope [°/s]
1	0.110	0.803
2	0.108	0.806
3	0.105	0.799

## Data Availability

The data used in this publication is GNSS/IMU navigation data, and cannot be made available under security restrictions.
